# Pathways of Non-enzymatic Lysine Acylation

**DOI:** 10.3389/fcell.2021.664553

**Published:** 2021-04-29

**Authors:** Tim Baldensperger, Marcus A. Glomb

**Affiliations:** ^1^Department of Molecular Toxicology, German Institute of Human Nutrition Potsdam-Rehbruecke (DIfE), Nuthetal, Germany; ^2^Institute of Chemistry, Food Chemistry, Martin-Luther-University Halle-Wittenberg, Halle, Germany

**Keywords:** advanced glycation endproducts, lysine acylation, reactive acyl-CoA species, α-dicarbonyls, acyl phosphates, aging

## Abstract

Posttranslational protein modification by lysine acylation is an emerging mechanism of cellular regulation and fine-tunes metabolic processes to environmental changes. In this review we focus on recently discovered pathways of non-enzymatic lysine acylation by reactive acyl-CoA species, acyl phosphates, and α-dicarbonyls. We summarize the metabolic sources of these highly reactive intermediates, demonstrate their reaction mechanisms, give an overview of the resulting acyl lysine modifications, and evaluate the consequences for cellular regulatory processes. Finally, we discuss interferences between lysine acylation and lysine ubiquitylation as a potential molecular mechanism of dysregulated protein homeostasis in aging and related diseases.

## Introduction

Acetylation of lysine side chains was first discovered in histone proteins by Phillips in 1963 (Phillips, 1963). In 1964 Allfrey linked the neutralization of positively charged lysine in chromatin *via* acetylation to weakened interaction with negatively charged DNA and hence increased gene expression (Allfrey et al., 1964). Prior to discovery of lysine acetyl transferases in 1995 (Kleff et al., 1995) it was observed that purified histones incubated with ^14^C-labeled acetyl-CoA incorporated the radioactivity without additional enzymes (Paik et al., 1970). Acetyl-CoA is formed by activation of the carboxylic acid function of acetate by acetyl-CoA synthetase under ATP consumption. Thus, the activated acetyl-CoA thioester can modify lysine residues non-enzymatically or is utilized by several lysine acetyl transferases for posttranslational modification of proteins. As a consequence of this combined enzymatic and non-enzymatic action acetylation was detected at thousands of proteins and emerged as a major regulatory mechanism in metabolism, aging, and disease (Ali et al., 2018).

**FIGURE 1:**
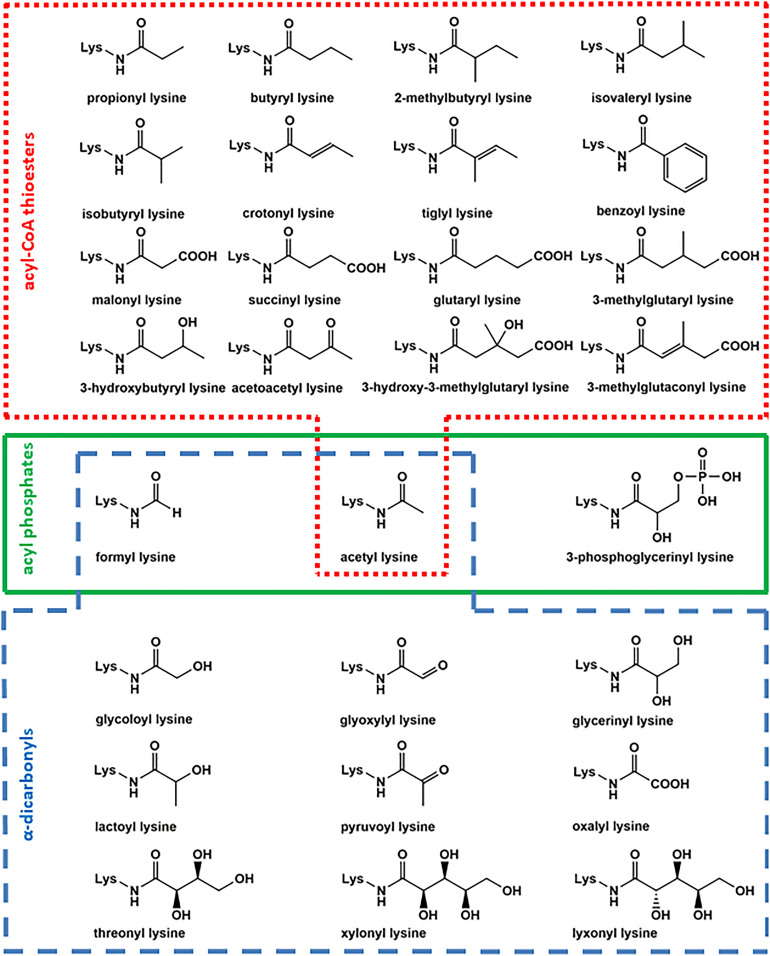
Overview of acyl lysine modifications. Several posttranslational modifications are formed by reaction of lysine with acyl-CoA thioesters (red box), acyl phosphates (green box), and α-dicarbonyls (blue box).

This fundamentally important process is paralleled by non-enzymatic reactions of several other acyl-CoA thioesters leading to a plethora of structurally related lysine acylation modifications (Figure 1; Simic et al., 2015; Wagner et al., 2017; Trub and Hirschey, 2018). Reactive acyl-CoA species like acetyl-CoA, succinyl-CoA, and malonyl-CoA are central intermediates in metabolism of carbohydrates, proteins, and lipids. Hence, they are highly abundant and reactive precursors for non-enzymatic protein modification (Gao et al., 2007). Acylation by acyl phosphates is an alternative pathway of non-enzymatic lysine modification in prokaryotes and eukaryotes (Jiang et al., 2007; Moellering and Cravatt, 2013; Weinert et al., 2013). As a third pathway formation of amide advanced glycation endproducts (amide AGEs) by α-dicarbonyls (Glomb and Pfahler, 2001; Henning et al., 2011; Baldensperger et al., 2018) was described in literature.

In the present mini review we give a short introduction into the different pathways of non-enzymatic lysine acylation and present an overview of the formed modifications, their precursors, and the consequences for regulatory processes. Finally, we discuss the potential cross-talk between acylation and ubiquitylation as a contributing factor of dysregulated protein homeostasis in aging. We hope this mini-review will raise awareness and facilitates future research in the emerging field of lysine acylation.

## Acylation by Acyl-CoA Thioesters

**FIGURE 2:**
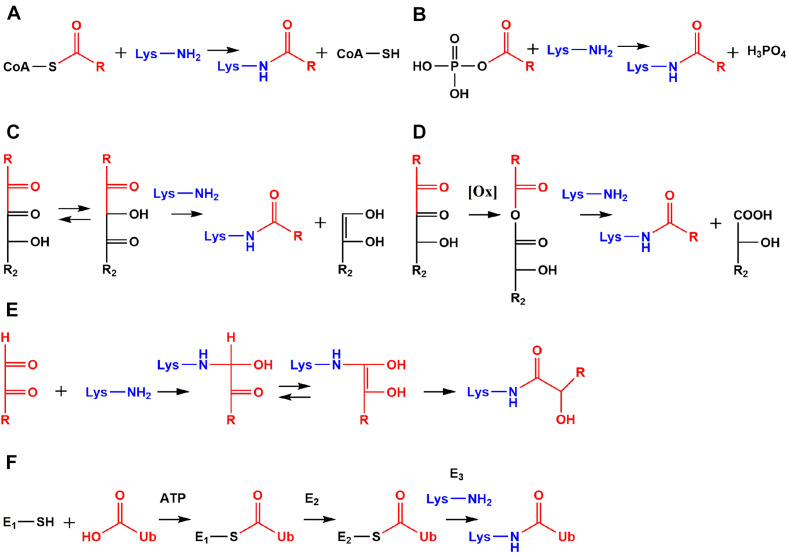
Comparison of non-enzymatic acylation mechanisms and ubiquitylation. Reactive species like acyl-CoA thioesters **(A)** and acyl phosphates **(B)** modify lysine residues non-enzymatically leading to protein modifications with an amide structure. Amide AGEs are formed by α-dicarbonyls *via* amine induced β-dicarbonyl fragmentation **(C)**, oxidative α-dicarbonyl fragmentation **(D)**, and isomerization **(E)**. Ubiquitin (Ub) is activated as a thioester by ubiquitin-activating enzyme (E1) under consumption of adenosine triphosphate (ATP). Activated Ub is transferred to an active site cysteine of ubiquitin-conjugating-enzymes (E2s). The high energy of the E2-Ub thioester is used by ubiquitin ligases (E3s) to form an amide bond between Ub and ε-amino functions of lysine residues **(F)**.

Acetylation is controlled by a plethora of different lysine acetyl transferases (“writers”) and lysine deacetylases (“erasers”) (Ali et al., 2018). However, acetyl-CoA and other acyl-CoA-thioesters are able to modify lysine residues independently of enzymes as well (Figure 2A). This non-enzymatic pathway was already described in the beginning of research about regulation of transcription by histone acetylation (Paik et al., 1970). It is currently considered as the major source of acetyl lysine in mitochondria, because of the low number of mitochondrial acetyl transferases, the high acetyl-CoA concentrations of up to 1.5 mM, and elevated pH of 8.0 facilitating nucleophilic attack of lysine at the CoA-thioester function (Wagner and Payne, 2013). Generally, the stoichiometry of acetyl lysine residues is below 1% (Baeza et al., 2016; Meyer et al., 2018) with a median stoichiometry of about 0.05% (Weinert et al., 2015). Acetyl-CoA is an important intermediate in the citric acid cycle, involved in catabolism of fatty acids, carbohydrates, and amino acids as well as biosynthesis of steroids and acetylcholine (Pietrocola et al., 2015).

Several hydrophobic derivatives of lysine acetylation (Figure 1) like aliphatic propionylation and butyrylation (Chen et al., 2007), unsaturated crotonylation and tiglylation (Tan et al., 2011; Baldensperger et al., 2019), branched-chain isobutyrylation, 2-methylbutyrylation, and isovalerylation (Baldensperger et al., 2019; Zhu et al., 2021), as well as aromatic benzoylation (Huang et al., 2018) have been reported in literature previously. Due to structural similarity, propionylation and butyrylation have effects comparable to acetylation in remodeling chromatin structure and activation of transcription (Kebede et al., 2017). The concentration of these aliphatic modifications is about a factor of 100 below lysine acetylation (Baldensperger et al., 2019). The precursor propionyl-CoA is involved in odd-chain fatty acid degradation, amino acid catabolism, and bile acid synthesis (Mosbach, 1972; Adeva-Andany et al., 2017; Wongkittichote et al., 2017). The homologous structure butyryl-CoA is mainly formed in lipid metabolism (Chen et al., 2007). Propionyl-CoA and butyryl-CoA are substrates of lysine acetyltransferases like KAT2B with tremendously reduced acylation rates compared to acetyl-CoA raising the question to which extend the respective modifications are formed enzymatically or non-enzymatically (Leemhuis et al., 2008).

Unsaturated modifications crotonylation and tiglylation are rather low abundant with concentrations three orders of magnitude below acetyl lysine (Baldensperger et al., 2019). However, several studies identified significant effects of crotonylation in important pathways like regulation of spermatogenesis and telomere maintenance (Liu et al., 2017; Fu et al., 2018). Classical “reader” proteins of acetylation such as YEATS domain and tandem plant homodomain have higher affinity for crotonylation compared to acetylation (Li et al., 2016; Xiong et al., 2016). Formation of crotonyl lysine is probably not exclusively non-enzymatic, because acetyltransferase p300 has extraordinary high crotonylation rates *in vitro* (Sabari et al., 2015). Crotonyl-CoA originates from metabolism of lysine or tryptophan (Rao et al., 2006) and tiglyl-CoA is an intermediate in metabolism of isoleucine (Gibson et al., 2000). Other CoA-thioesters formed in branched-chain amino acid metabolism are isobutyryl-CoA by valine (Roe et al., 1998), 2-methylbutyryl-CoA by isoleucine (Gibson et al., 2000) and isovaleryl-CoA by leucine catabolism (Mack et al., 2006). The corresponding acyl lysine modifications 2-methylbutyrylation and isovalerylation are concentrated in the same range as crotonylation. While this seems rather low abundant, their precursors are highly specific for branched-chain amino acid metabolism and may be important regulators of these pathways (Baldensperger et al., 2019). Benzoylation is currently the only known aromatic lysine acylation and closely linked to the food preservative sodium benzoate, which is activated as benzoyl-CoA by a still putative acyl-CoA synthetase (Huang et al., 2018).

The second prominent class of lysine modifications are acidic acylations (Figure 1) by malonylation (Peng et al., 2011), succinylation (Zhang et al., 2011), glutarylation (Tan et al., 2014), 3-hydroxy-3-methylglutarylation, 3-methylglutaconylation (Wagner et al., 2017), and 3-methylglutarylation (Anderson et al., 2017). Succinylation is one of the most abundant lysine acylations and reaches approximately 10–30% of acetylation levels, followed by malonylation with a factor of at least 10 below acetyl lysine concentration. The remaining acidic acylations are only found in trace amounts (Baldensperger et al., 2019). Malonyl-CoA is the key starting molecule in lipogenesis (Sanders and Griffin, 2016). The corresponding lysine modification is an efficient inhibitor of glyceraldehyde-3-phosphate dehydrogenase and, hence, the glycolytic flux (Nishida et al., 2015). The lysine succinylation precursor succinyl-CoA is a key citric acid cycle intermediate (Hirschey and Zhao, 2015). Elevated succinyl-CoA levels result in hypersuccinylation of succinate dehydrogenase and increases mitochondrial respiration (Park et al., 2013). Succinyl-CoA has an exceptional high non-enzymatic reactivity toward lysine acylation, because an intermediary cyclic anhydride with a 5-membered backbone is formed. This results in a 150-fold increase of reactivity compared to acetyl-CoA (Simic et al., 2015). Similar cyclic anhydrides with 6-membered backbones are formed by glutaryl-CoA, 3-hydroxy-3-methylglutaryl-CoA, and 3-methylglutaconyl-CoA (Wagner et al., 2017). Glutaryl-CoA is a precursor of crotonyl-CoA in the oxidative degradation of lysine and tryptophan (Rao et al., 2006), 3-methylglutaconyl-CoA is originating from leucine catabolism and is a precursor of 3-methylglutaryl-CoA and 3-hydroxy-3-methylglutaryl-CoA (Mack et al., 2006). The corresponding acylation modifications are regulators of leucine metabolism and insulin secretion (Anderson et al., 2017).

Alternatively, 3-hydroxy-3-methylglutaryl-CoA as well as precursors of recently discovered lysine 3-hydroxybutyrylation (Xie et al., 2016) and acetoacetylation (Baldensperger et al., 2019) are formed in ketone body metabolism (Grabacka et al., 2016). Both modifications are low abundant, but the high specificity of their precursors for ketone body metabolism makes them valuable marker and regulatory structures. Especially supplementation of β-hydroxybutyrate and resulting 3-hydroxybutyrylation of lysine, also known as β-hydroxybutyrylation, is a frequently reported mechanism with diverse beneficial effects like mimicking calorie restriction and reducing oxidative stress (Shimazu et al., 2013; Xie et al., 2016; Izuta et al., 2018). The formation of 3-hydroxybutyrylation is enzymatically facilitated by p300 and CBP (Liu et al., 2019; Huang et al., 2021).

## Acylation by Acyl Phosphates

A major pathway of lysine acetylation in bacteria like *Escherichia coli* (*E. coli*) is non-enzymatic modification by acetyl phosphate (Figure 2B; Weinert et al., 2013; Kuhn et al., 2014). Acetyl phosphate is a central intermediate in bacterial energy metabolism (Enjalbert et al., 2017). This highly reactive mixed anhydride is detrimental for proteolysis (Mizrahi et al., 2006) and stress response in *E. coli* (Bouché et al., 1998). Recently, formation of acetyl phosphate was detected in human mitochondria for the first time, but relevance for human metabolism and mitochondrial acetylation is still unclear and needs to be addressed in future research (Xu et al., 2018). However, acylation by acyl phosphates is definitively not limited to prokaryotes, but is relevant in higher eukaryotes like humans as well. For example, the stable 3-phosphoglycerinyl lysine modification is formed non-enzymatically by 1,3-bisphosphoglycerate, which is an intermediate in glycolysis. The modification was found at several sites of enzymes involved in glycolysis, inhibited their activity, and was proposed as an energy feedback mechanism (Moellering and Cravatt, 2013). Another acyl phosphate is formyl phosphate, which is proposed to be generated by 5′-oxidation of the deoxyribose phosphate backbone in DNA (Dedon et al., 1992) to give formyl lysine. In this context *in vivo* oxidation of DNA catalyzed by neocarzinostatin as well as formaldehyde treatment resulted both in elevated lysine formylation (Jiang et al., 2007; Edrissi et al., 2013). The biological significance of this modification is currently poorly understood, but our own research indicated rather high abundance in various tissues (Baldensperger et al., 2019) and a strong accumulation at histone proteins in the aging process (Baldensperger et al., 2020).

## Acylation by α-Dicarbonyls

Last but not least, α-dicarbonyls are important precursors of non-enzymatic lysine acylation in the context of glycation leading to formation of amide advanced glycation endproducts (amide AGEs) (Henning et al., 2011; Henning and Glomb, 2016). A class of reactive α-dicarbonyls called deoxyglucosones is generated as intermediates in the Maillard reaction (Bruhns et al., 2018). The best studied structures of this class are 3-deoxyglucosone (3-DG), 1-deoxyglucosone (1-DG), and glucosone. While literature heavily focuses on metabolic sources and modification by 3-DG (Rabbani and Thornalley, 2015), the reductone structures 1-DG and glucosone have a much higher reactivity (Glomb et al., 2001). An important α-dicarbonyl originating from irreversible ascorbic acid oxidation is 2,3-diketogulonic acid (Dewhirst and Fry, 2018). Among the most reactive α-dicarbonyls are short-chained molecules like glyoxal and methylglyoxal, because their structure is not stabilized as cyclic hemiacetals. Glyoxal and methylglyoxal are both formed during Maillard-induced carbohydrate degradation, but several additional metabolic pathways are important sources like lipid peroxidation for glyoxal and degradation of triosephosphates as a side reaction of glycolysis for methylglyoxal (Rabbani and Thornalley, 2015).

Amide AGEs are formed by three distinct mechanisms *in vivo*: amine induced β-dicarbonyl fragmentation (Figure 2C), oxidative α-dicarbonyl fragmentation (Figure 2D), and isomerization of short-chained α-dicarbonyls (Figure 2E; Henning and Glomb, 2016). The amine induced β-dicarbonyl fragmentation of 1-DG is an alternative source of acetyl lysine next to modification by acetyl-CoA and acetyl phosphate (Smuda et al., 2010). Isomerization of 1-DG leads to 1-deoxy-2,4-hexodiulose and nucleophilic attack of lysine at the C2 carbonyl function initiates β-dicarbonyl fragmentation and acetyl lysine formation. Alternatively, glycerinyl lysine is formed by attack of lysine at the C4 carbonyl function of 1-deoxy-2,4-hexodiulose. The isomerization can proceed further and affects the complete 1-DG backbone until 1-deoxy-4,6-hexodiulose is generated, which is one of the precursors of formyl lysine formation by β-dicarbonyl fragmentation (Smuda et al., 2010). On the other hand, glucosone produced twice as much formyl lysine compared to 1-DG degradation *in vitro* (Henning et al., 2011).

Alternative to β-dicarbonyl scission oxidative α-dicarbonyl fragmentation efficiently leads to amide AGEs in presence of activated oxygen species. In the first step, singlet oxygen attacks a carbonyl function and is incorporated into the molecule as a hydroperoxide followed by one-electron transfer reactions. In a Baeyer–Villiger type rearrangement the hydroperoxide is transformed into an anhydride intermediate. This anhydride is cleaved by lysine into formyl lysine and the carboxylic acid counterpart. The oxidative α-dicarbonyl fragmentation of 2,3-diketogulonic acid results in formation of oxalyl and threonyl lysine. While 50% of total oxalyl lysine was formed by β-dicarbonyl fragmentation as indicated by incorporation of isotopically labeled oxygen, threonyl lysine was exclusively generated by oxidative α-dicarbonyl fragmentation of 2,3-diketogulonic acid. A special case is the formation of isomers xylonyl and lyxonyl lysine by decarboxylation of the diketogulonic acid hemiaminal (Smuda and Glomb, 2013).

Isomerization of glyoxal and methylglyoxal after nucleophilic attack of lysine at the aldehyde function yields glycoloyl and lactoyl lysine, respectively (Glomb and Pfahler, 2001; Jost et al., 2018). Additional pathways of lysine lactoylation are β-dicarbonyl fragmentation of 1-DG isomerization product 1-deoxy-3,5-hexodiulose (Smuda et al., 2010) and the non-enzymatic acyl transfer verified from lactoyl glutathione or recently proposed from lactoyl-CoA thioester, which are controversially discussed (Di Zhang et al., 2019; Gaffney et al., 2020; Varner et al., 2020). The novel α-oxoamide AGEs glyoxylyl and pyruvoyl lysine are formed by oxidation of the central enaminol intermediates in the glyoxal and methylglyoxal isomerization cascades, respectively (Baldensperger et al., 2018).

Amide AGEs have been previously quantitated in various diseases and different matrices. For example concentrations increased in cataractous eye lenses (Smuda et al., 2015), blood of uremic patients (Henning et al., 2011), collagen stiffening in aging (Jost et al., 2018), and aging of liver proteins (Baldensperger et al., 2018, 2020). In a study on human lens crystallins glycation of most amide AGEs correlated positively with age with two exceptions (Smuda et al., 2015). Acetyl lysine basically remained at constant levels, which are highly regulated due to above discussed pathways. However, unexpectedly threonyl lysine resulting from non-enzymatic degradation of ascorbic acid behaved the same and, in this case, no alternative enzymatic formation nor degradation mechanisms are known so far.

## Discussion

Lysine acylation is an emerging regulatory mechanism with a huge impact on various cellular processes (Table 1). Research focuses on biological functions of these recently discovered posttranslational modifications and novel insights are constantly published. However, the particularly interesting field of possible cross-talk with other lysine modifications like ubiquitylation is currently poorly understood. Enzymatic ligation of ubiquitin to proteins was originally identified as a pivotal mark for degradation by the proteasomal system (Hershko et al., 1980), but nowadays ubiquitylation is also linked to distinct functions like cellular signaling and quality control of the genome (Schwertman et al., 2016; Cockram et al., 2021). Ubiquitin is a small protein consisting of 76 amino acids and was discovered “ubiquitously” in tissues of eukaryotic organisms (Goldstein et al., 1975). In the initial step of ubiquitylation (Figure 2F) the carboxylic acid function of glycine at the ubiquitin C-terminus is activated by the ATP-consuming ubiquitin-activating enzyme (E1). An intermediary ubiquitin adenylate is formed and reacts with a cysteine residue of E1 leading to formation of a reactive thioester bond between E1 and ubiquitin. The activated ubiquitin thioester is transferred to an active site cysteine of ubiquitin-conjugating enzymes (E2s). The high energy stored in the thioester bond of the E2-ubiquitin complex is used by ubiquitin ligases (E3s) for protein ubiquitylation *via* amide bond formation with ε-amino groups of lysine residues. Hence, both acylation and ubiquitylation use reactive thioester intermediates to modify ε-amino functions of lysine residues. But these are not the only similarities. At least in the case of acetylation (Hershko et al., 1980; L’Hernault et al., 1985) both modifications have complex sets of regulatory enzymes including “writers” (ubiquitin ligases vs. lysine acetyl transferases), “readers” (ubiquitin binding domains vs. bromodomains), and “erasers” (deubiquitinating enzymes vs. lysine deacetylases) (Hershko and Ciechanover, 1998; Reyes-Turcu et al., 2009; Husnjak and Dikic, 2012; Ali et al., 2018).

**TABLE 1:** Acyl lysine modifications and their biological functions reported in literature.

**Modification**	**Biological function**
Formyl lysine	Increase in oxidative stress (Jiang et al., 2007) Increase in formaldehyde exposition (Edrissi et al., 2013) Increase in aging (Baldensperger et al., 2020)
Acetyl lysine	Major regulator of various cellular processes (Ali et al., 2018)
Propionyl lysine	Activation of transcription (Kebede et al., 2017) Binding of bromodomain (Flynn et al., 2015) Sensor for propionyl-CoA (Garrity et al., 2007)
Butyryl lysine	Activation of transcription (Kebede et al., 2017) Binding of bromodomain (Flynn et al., 2015)
Isobutyryl lysine	Activation of transcription (Zhu et al., 2021)
Crotonyl lysine	Activation of transcription (Tan et al., 2011) Regulation of spermatogenesis (Liu et al., 2017) Maintenance of telomers (Fu et al., 2018) Binding of bromodomains (Flynn et al., 2015)
Benzoyl lysine	Activation of transcription (Huang et al., 2018)
Malonyl lysine	Inhibition of glycolysis (Nishida et al., 2015) Induction of inflammation (Galván-Peña et al., 2019)
Succinyl lysine	Activation of mitochondrial respiration (Park et al., 2013) Activation of chaperone activity (Nandi et al., 2019) Activation of transcription (Smestad et al., 2018) Inhibition of lysosomal degradation (Li et al., 2020)
3-Hydroxybutyryl lysine	Inhibition of p53 (Liu et al., 2019) Activation of transcription and starvation response (Xie et al., 2016)
3-Hydroxy-3 methylglutaryl lysine	Inhibition of leucine catabolism (Anderson et al., 2017) Reduction of insuline sensitivity (Anderson et al., 2017)
3-Phosphoglycerinyl lysine	Inhibition of glycolysis (Moellering and Cravatt, 2013)
Glycoloyl lysine	Increase in aging (Smuda et al., 2015; Baldensperger et al., 2018, 2020)
Glyoxylyl lysine	Increase in aging and liver cirrhosis (Baldensperger et al., 2018)
Glycerinyl lysine	Increase in aging (Smuda et al., 2015)
Lactoyl lysine	Increase in aging (Smuda et al., 2015; Baldensperger et al., 2018, 2020)
Pyruvoyl lysine	Increase in aging and liver cirrhosis (Baldensperger et al., 2018)
Oxalyl lysine	Increase in aging (Smuda et al., 2015)

Ubiquitylation and acetylation are among the most abundant posttranslational modifications and a strong overlap of ubiquitylation and acetylation sites was detected in the human proteome by mass spectrometry (Wagner et al., 2011). This results in competition and a possible “cross-talk” between lysine acetylation and ubiquitylation. While ubiquitylation is an important marker for proteasomal degradation (Hershko et al., 1980), acetylation is a marker of proteins with long turnover times like cytoskeletal α-tubulin (L’Hernault et al., 1985). For instance, stability of tumor suppressor p53 is increased by acetylation counteracting ubiquitylation (Caron et al., 2005) and direct acetylation of ubiquitin is an inhibitor of polyubiquitylation (Ohtake et al., 2015). Very recently, the inhibition of ubiquitylation by succinylation was demonstrated for lactate dehydrogenase resulting in higher cell proliferation, invasion, and migration in gastric cancer (Li et al., 2020).

A central hallmark of aging is severely impaired protein homeostasis in aged organisms (López-Otín et al., 2013). The amount of ubiquitin and ubiquitylated protein aggregates is increasing in the aging process and related diseases (Li et al., 2008; Kevei and Hoppe, 2014). In case of protein acetylation studies report mixed results of increase, decrease, and no significant changes in aging (Feser and Tyler, 2011; Kim-Ha and Kim, 2016; Wang et al., 2018; Baldensperger et al., 2020). As demonstrated above the enzymatic processes of lysine ubiquitylation and acetylation are paralleled by non-enzymatic acylation *via* acyl CoA-thioesters, acyl phosphates, and α-dicarbonyls. In contrast to enzymatic acetylation we unequivocally proved that many non-enzymatic acyl lysine modifications are accumulating at several proteins in the aging process (Baldensperger et al., 2020). Consequently, we postulate non-enzymatic lysine acylation as a key mechanism in aging and impaired protein homeostasis. We hope this review will encourage more researchers to test this hypothesis and raise awareness of these novel lysine modifications.

## Author Contributions

TB conceptualized and wrote the manuscript. MG conceptualized and reviewed the manuscript. Both authors contributed to the article and approved the submitted version.

## Conflict of Interest

The authors declare that the research was conducted in the absence of any commercial or financial relationships that could be construed as a potential conflict of interest.
